# Purification, characterization, gene cloning and expression of GH-10 xylanase (*Penicillium citrinum* isolate HZN13)

**DOI:** 10.1007/s13205-016-0489-4

**Published:** 2016-08-13

**Authors:** Zabin K. Bagewadi, Sikandar I. Mulla, Harichandra Z. Ninnekar

**Affiliations:** Department of Biochemistry, Karnatak University, Dharwad, Karnataka 580 003 India

**Keywords:** Xylanase, *Penicillium citrinum*, Purification, Characterization, Cloning and expression

## Abstract

An extracellular thermostable xylanase (Xyl-IIb) produced by *Penicillium citrinum* isolate HZN13 was purified to homogeneity using DEAE-Sepharose, Sephadex G-100 and Bio-Gel P-60 chromatography with specific activity of 6272.7 U/mg and 19.6-fold purification. The purification revealed the occurrence of multiple forms of xylanases (Xyl-I, Xyl-IIa, Xyl-IIb and Xyl-III). The molecular mass of highly purified Xyl-IIb was ~31 kDa with SDS-PAGE. The enzyme was cellulase-free, thermostable (55–75 °C) and acidophilic (3.5–5.0). It was activated by Ca^2+^, Ba^2+^, DTT and β-mercaptoethanol, whereas inhibited by Hg^2+^, Pb^2+^, Ni^2+^ and *p*-CMB. Purified Xyl-IIb exhibited highest specificity toward birchwood and oat spelts xylan. Kinetics of Xyl-IIb revealed a *K*_m_ of 10 mg/ml and 16.7 mg/ml and *V*_max_ of 9523g and 15,873 U/mg with birchwood and oat spelts xylan, respectively, indicating high affinity toward birchwood xylan. The xylanase (Xyl-IIb) belongs to glycosyl hydrolase (GH) family 10 based on conserved regions. Xylanase-encoding gene (*xynB*) consists of 1501 bp with an open reading frame of 264 bp which was predicted to encode a protein having 87 amino acids and shared homology with endo-1,4-beta-xylanase (*xynB*) gene from *Penicillium citrinum*. Cloned *xynB* gene was expressed in *E. coli* BL21 (DE3) with xylanase activity (80 U/mg) and confirmed to be GH-10 Xyl-IIa based on molecular mass (~40 kDa). These properties of xylanase make it promising for their applications in biofuel industries.

**Electronic supplementary material:**

The online version of this article (doi:10.1007/s13205-016-0489-4) contains supplementary material, which is available to authorized users.

## Introduction

Xylan, the structural polysaccharide, is a heteroglycan composed of a backbone of β-(1,4)-linked d-xylopyranosyl residues. Different substituents like l-arabinofuranose, d-glucuronic acid, and 4-*o*-methyl-d-glucuronic acid are attached at 2′ and 3′ positions (Wakiyama et al. 2008). The complete hydrolysis of xylan requires the synergistic action of different xylanolytic enzymes (xylanase, β-xylosidase, α-l-arabinofuranosidase, α-d-glucuronidase and acetyl xylan esterase) (Beg et al. 2001). Among them, xylanases (EC 3.2.1.8) deserve special attention as they degrade major hemicellulosic polysaccharides by catalyzing the hydrolysis of xylopyranosyl linkages of β-1,4-xylan (Zhang et al. 2011). Xylanases are used in biobleaching of pulp in paper industry, bioconversion of lignocellulosic waste into fermentative products, improvement of animal feed quality and clarification of fruit juices and wines (Fang et al. 2010; Juturu and Wu 2012; Maity et al. 2012). Xylanases are produced from a variety of agricultural wastes. Sweet sorghum bagasse is cheap, widely distributed, available in plenty and serves as a better feedstock for xylanase production as it is non-food based agricultural residue having high carbohydrate content. Sweet sorghum is unique among the other crops as feedstock for renewable energy due to high photosynthetic activity, thereby generating high biomass yields and also survives water deficit conditions, abiotic stresses, heat and dry environments, water logging, salinity, alkalinity and other constraints (Teshome and Charles 2014). Highly active, thermostable xylanases are useful for industrial applications. Xylanases are widely distributed in bacteria, fungi and actinomycetes. However, fungal sources are most desirable for enzyme production due to their higher stability even under harsh environmental conditions (Moretti et al. 2012). Several filamentous fungi, which exhibit high xylan-degrading capabilities and thus producing multiple xylanases, have been studied (Liao et al. 2012). *Aspergillus* sp. and *Trichoderma* sp. are the most dominant among the high xylanase producers and commonly employed at an industrial level. Other strains like *Penicillium oxalicum* GZ-2 have been reported (Liao et al. 2014). However, there are few reports on the occurrence of multiple xylanases from *Penicillium* species (Liao et al. 2015). Current research on exploring new xylanases still exists (Miao et al. 2015). Based on the sequence similarities of amino acid, xylanases are classified into glycosyl hydrolase (GH) families 10 (GH10) and 11 (GH11) (Guillen et al. 2010), but other GH families like 5, 8 and 43 are also found (http://www.cazy.org). GH 10 contains xylanases of high molecular mass (>30 kDa) with a (β/α)_8_ barrel structure and acidic *pI* values. GH11 are the low-molecular-weight endoxylanases (Henrissat and Bairoch 1996) which are divided into alkaline *pI* and acidic *pI* xylanases. Moreover, these enzymes within each family do not necessarily share similar enzymatic properties (Jørgensen et al. 2003). Many xylanases from bacteria and fungi have been cloned and characterized. Several xylanases genes from *Penicillium* sp. have been cloned and expressed, such as *Penicillium citrium* XynA, *Penicillium* *funiculosum* XYNC, *Penicillium* sp. strain 40 XynA, *Penicillium purpurogenum* XynB, and *Penicillium janthinellum* (Liu et al. 2010). There are very few reports on *xynB* gene from *Penicillium citrinum* (Ahmed et al. 2009). In this paper, we report the occurrence of multiple xylanases as well as purification, properties, gene cloning and sequencing and expression of GH-10 xylanase from *Penicillium citrinum* isolate HZN13.

## Materials and methods

### Chemicals, strains and vectors

Chemicals, substrates and column matrix used were purchased from Sigma-Aldrich (USA), Merck (USA) and Bio-Rad (France). Sweet sorghum stalks were provided by University of Agricultural Sciences, Dharwad. *E. coli* DH5α and *E. coli* BL21 (DE3) were used for gene cloning, sequencing and expression studies, respectively. pGEM-T and pGEX-4T-1 were used as cloning and expression vectors, respectively.

### Xylanase production conditions

A fungal strain previously isolated in our laboratory from forest soil and identified as *Penicillium citrinum* isolate HZN13 was used in the present study. Xylanase production by *Penicillium citrinum* isolate HZN13 was carried out by solid state fermentation (SSF) using alkali-pretreated sweet sorghum bagasse in 250-ml Erlenmeyer flasks in modified Mandels–Weber medium containing (g/l) urea 0.3; ammonium sulfate 1.4; KH_2_PO_4_ 0.3; CaCl_2_ 0.3; MgSO_4_·7H_2_O 0.3; yeast extract 1.0; lactose 10; and (mg/l) FeSO_4_·7H_2_O 5.0; MnSO_4_·7H_2_O 1.6; ZnSO_4_·7H_2_O 1.4; CoCl_2_ 2; Tween-80 0.1 % (pH 4) (Bagewadi et al. 2016). The clear supernatant obtained was used as a source of crude enzyme for further purification.

### Enzyme assay and protein determination

Xylanase activity was determined using the modified method of Bailey et al. (1992) with 1 % (w/v) birchwood xylan substrate. Cellulase activity was estimated using 1 % (w/v) carboxymethyl cellulose (CMC) in sodium acetate buffer (pH 5) at 40 °C for 30 min using modified method (Eveleigh et al. 2009). The reducing sugars released from the above reactions were determined according to Miller (1959) by dinitrosalicylic acid (DNS) method. One unit (U) of enzyme activity was defined as the amount of enzyme that released 1 μmol of the reducing sugars (glucose or xylose equivalent) per minute under standard assay conditions. Specific activity was expressed as units per milligram of protein. The concentrations of soluble proteins were estimated by BCA protein assay kit (Mulla et al. 2016). All assays were performed in triplicate, and the results are presented as mean ± standard deviation.

### Enzyme purification

Xylanase produced by *Penicillium citrinum* isolate HZN13 was purified by methods described previously (Bagewadi et al. 2016). Briefly, all purification steps were carried out at 4 °C unless otherwise specified. The crude enzyme was fractionated by (NH_4_)_2_SO_4_ (80 % saturation) and dialyzed (50 mM sodium citrate buffer, pH 4.0). The enzyme solution was concentrated by ultrafiltration system (Amicon, USA) with a 10-kDa cut-off membrane and lyophilized. The lyophilized enzyme was purified by DEAE-Sepharose and Sephadex G-100 columns with 50 mM sodium citrate buffer (pH 4.0) as a mobile phase. Further purification was carried out with Bio-Gel P-60 column (3.4 × 110 cm) pre-equilibrated and eluted with 50 mM sodium citrate buffer (pH 4.0) at a flow rate of 0.5 ml/min. Fractions containing xylanase activity were pooled, concentrated and used for further characterization.

### SDS-PAGE, zymogram analysis and amino acid sequencing

The purified enzyme was subjected to sodium dedocyl sulfate-polyacrylamide gel electrophoresis (SDS-PAGE) according to the method described previously (Laemmli 1970). Protein bands on the gel were visualized by staining with Coomassie Brilliant Blue R-250. For zymogram analysis, a native PAGE electrophoresis was carried out with polyacrylamide gel (10 %) at 4 °C using TBE buffer (89 mM Tris, 2 mM EDTA, and 89 mM boric acid). After electrophoresis, the gel was soaked in 50 mM sodium citrate buffer (pH 4) containing 1 % birchwood xylan and incubated for 30 min at 45 °C to detect xylanase activity and then stained with Congo red solution (2 mg/ml) for 15 min at room temperature. The gel was washed with 1 M NaCl solution and transferred to 5 % (v/v) acetic acid solution. Clear areas in a dark red background indicated xylanase activity (Driss et al. 2012). For amino acid sequencing, the enzyme was digested with V8 protease (Wakiyama et al. 2008). The N-terminal amino acid sequences of the protein and resulting peptide fragments were identified using protein sequencing system (Shimadzu, Japan).

### Biochemical characterization of purified xylanase

The optimum pH of the purified xylanase was determined using different buffers described in previous study by Bagewadi et al. (2016). The enzyme stability in a pH range of 3.5–5.0 was determined after incubation for 3 h. The optimum temperature for enzyme activity was determined between 20 and 85 °C. The thermostability of xylanase was determined between 55 and 75 °C for 3 h at pH 4.0. The effect of various metal ions, thiol reagents, oxidizing agents, metal chelators and detergents at different concentrations of 2, 5 and 10 mM on the enzyme activity has been studied. The enzyme stability in the presence of various organic solvents was also evaluated. Substrate specificity of xylanase was determined using different substrates like 1 % birchwood xylan, oat spelts xylan, CMC, microcystalline cellulose, chitin, cellobiose, starch, filter paper, pectin, PNP-α-galactopyranoside, PNP-glucopyranoside and PNP-cellobioside. The kinetics parameters *K*_m_ and *V*_max_ of purified Xyl-IIb were determined with birchwood and oat spelts xylan substrate with a concentration range of 2–20 mg/ml by Lineweaver–Burk double reciprocal plot.

### Cloning of xylanase gene (xynB) from *Penicillium citrinum* isolate HZN13

The xylanase gene from *Penicillium citrinum* isolate HZN13 was amplified using the cDNA, reverse-transcribed from RNA (template). The following gene specific primers: forward primer: cg**gaattc**atatggttcaaatcaag and reverse primer: cg**gaattc**aaaggcctctagag (bold letters indicate Eco RI restriction sites) were designed based on the conserved sequences revealed by sequence alignment and comparative analysis of *Penicillium citrinum* xylanase homologous genes from the GenBank database. Reverse transcription-polymerase chain reaction (RT-PCR) (Eppendorf, Germany) was performed in 50 μl reaction mixtures containing synthesized cDNA under the following conditions: 40 cycles of 1 min denaturation at 94 °C, 1 min annealing at 48 °C, and 1 min amplification at 72 °C. The amplified products (1.5 kb) were gel-purified, ligated into the pGEM-T vector and transformed into *E. coli* DH5α. Screening for xylanase-positive clones was carried out on 1 % birchwood xylan-LB agar plates by Congo red plate assay. The clones with clear zones on the plates harbored xylanase gene.

### Sequence analysis and expression of xynB gene

Recombinant plasmids (pGEM-T-*xynB*) were isolated from xylanase-positive clones, and *xynB* gene sequencing was carried out using the Big Dye Terminator cycle sequencing kit (V3.1, Applied Biosystems, USA) according to the manufacturer’s protocol and analyzed in a DNA Analyzer (3730 DNA Analyzer, Applied Biosystems, USA). Two independent PCR products were sequenced in both directions to confirm the sequence. Sequence analysis was carried out using BLASTn program (http://www.ncbi.nlm.nih.gov/). Gene sequences were aligned using Clustal*W* (http://www.ebi.ac.uk/Tools/msa/clustalo/) (Ewing et al. 1998). Phylogenetic relationships were established using *xynB* gene sequence of isolated fungal strain (*Penicillium citrinum* isolate HZN13) with other related gene sequences (Tamura et al. 2013) by the neighbor-joining (NJ) method using MEGA6 software. The number at nodes showed the bootstrap values obtained with 1000 resampling analyses. The nucleotide sequence of gene was analyzed using the National Center for Biotechnology Information (NCBI) Open Reading Frame (ORF) Finder tool. Multiple sequence alignment of the putative amino acid sequence of xylanase with other related xylanases was carried out using the Clustal*W* software.

Furthermore, *xynB* gene-amplified product was sub-cloned into pGEX-4T-1 expression vector (designated as pGEX-4T-1-*xynB*) and was expressed in *E. coli* BL21 (DE3). The expression of xylanase was examined by detecting xylanase activity from the culture broth of the recombinant strain. To induce xylanase production, the *E. coli* BL21 (DE3) transformants were grown in an LB-ampicillin medium at 37 °C. When absorbance at 600 nm reached 0.5, isopropyl-β-D-thiogalactopyranoside (IPTG) was added at the final concentration of 1 mM, and the transformants were grown for an additional 4 h at 25 °C. Cells were harvested, resuspended in PBS solution (10 mM NaH_2_PO_4_, 150 mM NaCl, pH 7.2), and lysed by sonication. The supernatant was used for xylanase assay, and expression of *xynB* Gene was checked by SDS-PAGE. The nucleotide sequence of *xynB* gene from *Penicillium citrinum* isolate HZN13 was deposited in the GenBank with the Accession No. KU298274.

## Results and discussion

### Purification of xylanase

The properties of xylanase from *Penicillium citrinum* isolate HZN13 were assessed by purification by (NH_4_)_2_SO_4_ precipitation, ultrafiltration, DEAE-Sepharose, Sephadex G-100 and Biogel P-60 chromatography. During ultrafiltration, maximum xylanase activity was detected in the retentate and used for subsequent purification. Three major peaks (Xyl-I, Xyl-II and Xyl-III) exhibiting xylanase activities were obtained from the column chromatography using DEAE-Sepharose matrix. Xyl-I was not retained on the column and was eluted out first, whereas Xyl-II and Xyl-III were retained onto the column which were eluted out with an established NaCl gradient as shown in Fig. S1A (Supplementary information, SI). However, Xyl-II showed higher activity (49,600 U) which was further purified by Sephadex G-100. This purification step resulted in two peaks designated as Xyl-IIa and Xyl-IIb containing proteins with xylanase activity (Fig. S1B). Xyl-IIb fraction obtained from chromatography with Sephadex G-100 matrix was re-purified by column chromatography using Bio-Gel P-60 matrix where Xyl-IIb was eluted out as a single sharp homogenous peak, as revealed from the chromatogram, indicating high purity (Fig. S1C). The yield and fold purification of Xyl-IIb were 11.5 % and 19.60, respectively, with specific activity of 6272.7 U/mg protein as shown in Table 1. The purification reveals the presence of multiple isoforms of xylanase.

**Table 1:** Purification summary of xylanase from *Penicillium citrinum* isolate HZN13

Purification steps	Total protein (mg)	Total activity (U) × 10^3^	Specific activity (U/mg)	Yield (%)	Fold purification
Crude extract	750	240	320	100	1
Ammonium sulfate	200	104	520	43.34	1.62
Fractionation (80 %)					
Ultrafiltration	102	66	647	27.50	2.02
DEAE-Sepharose					
Xyl-I	18.90	47.70	2523.80	19.88	7.89
Xyl-II	25.60	49.60	1937.50	20.67	6.05
Xyl-III	4.40	17.20	3909.10	7.17	12.21
Sephadex G-100					
Xyl-IIa	4.80	36	7500	15	23.43
Xyl-IIb	10.50	46.90	4466.70	19.54	13.96
Bio-Gel P-60					
Xyl-IIb	4.40	27.60	6272.70	11.50	19.60

### Molecular mass determination, zymogram and amino acid sequence analysis

**Fig. 1:**
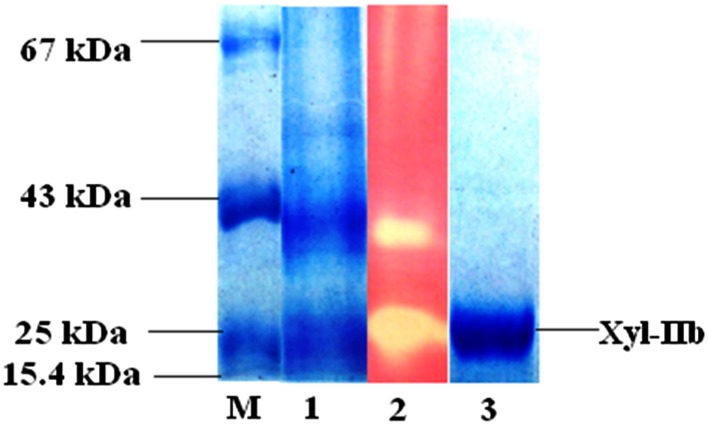
SDS-PAGE and zymogram analysis. *Lane M* standard molecular weight markers [RNase (15.4 kDa), chymotrypsin (25.0 kDa), ovalbumin (43.0 kDa) and BSA (67.0 kDa)]. *Lane 1* purified xylanase (Xyl-IIa and Xyl-IIb) on Sephadex G-100. *Lane 2* activity of Xyl-IIa and Xyl-IIb coincident with protein bands. *Lane 3* purified Xyl-IIb by Bio-Gel P-60 chromatography

The highly purified Xyl-IIb showed a single protein band on SDS-PAGE (Lane 3) with molecular weight of ~31 kDa, and Xyl-IIa purified by Sephadex G-100 showed a protein band of molecular mass of ~40 kDa (Lane 1) as shown in Fig. 1. The protein bands were examined for their ability to hydrolyze the xylan incorporated into the gel. Three distinct bands and a smear in the region of high molecular weight showing xylanase activity were detected in the gel with crude extract. The smear region disappeared as purification proceeded, indicating the separation of higher molecular weight proteins (xylanases) during chromatography. The zymogram analysis revealed the fact that multiple xylanases of GH-10 family with high molecular masses were produced by *Penicillium citrinum* isolate HZN13. As reported in the literature (Henrissat and Bairoch 1996), xylanases with high molecular mass (>30 kDa) belong to GH-10 family. The purified Xyl-IIb was cellulase-free with no measurable activities with CMC and only showed activity with birchwood and oat spelts xylans as the substrates. Xylanases with differing molecular mass from different organisms have been studied, such as 19 and 14 kDa from *Trichoderma inhamatum* (Silva et al. 2015) and 25 kDa from *Penicillium ramulosum* N1 (Lin et al. 2015). Multiple xylanase forms have also been studied in *A. fumigates* Z5 (Miao et al. 2015) and *Penicillium oxalicum* GZ-2 (Liao et al. 2015). Edman degradation method for N-terminal sequencing was unsuccessful most likely due to blockage of N-terminal amino acid. Such blockage is well reported in GH-10 xylanase family (Wakiyama et al. 2008). However, the N-terminal sequence of peptide fragment was determined: NSMKWDATER. Protein database search for the peptide sequence revealed close homology to GH-10 family of xylanases.

### Biochemical characterization of purified xylanases

#### Effect of pH and temperature

**Fig. 2:**
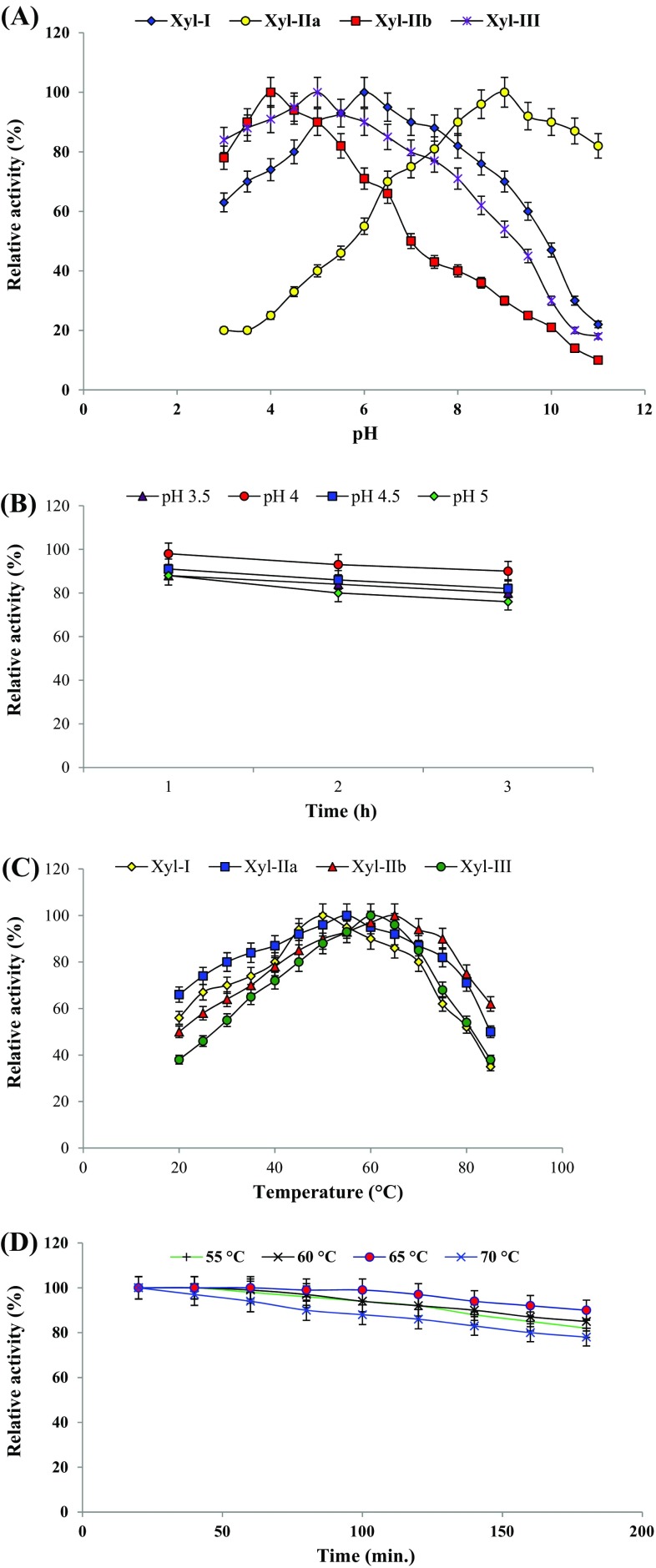
Effect of pH on purified xylanase (Xyl-I, Xyl-IIa, Xyl-IIb and Xyl-III) (**a**), pH stability of Xyl-IIb (**b**), effect of temperature (°C) on purified xylanase (Xyl-I, Xyl-IIa, Xyl-IIb and Xyl-III) (**c**), temperature stability of Xyl-IIb (**d**). *Data values* represent average of triplicates and *error bars* represent standard deviation

Multiple xylanases revealed different properties. Xyl-I, Xyl-IIa, Xyl-IIb and Xyl-III showed optimum activity at pH 6, 9, 4 and 5, respectively (Fig. 2a). Highly purified Xyl-IIb was remarkably active in the acidic pH range of 3.5-5.0. 80, 90, 82 and 76 % of Xyl-IIb activities were retained at pH 3.5, 4, 4.5 and 5, respectively, after 3 h indicating its stability at acidic pH (Fig. 2b). Xyl-I, Xyl-IIa, Xyl-IIb and Xyl-III showed optimum activity at temperatures 50, 55, 65 and 60 °C, respectively (Fig. 2c). Xyl-IIb was found to be highly stable at 55–75 °C (Fig. 2d). Enzymatic stability at their optimum conditions is needed for catalytic efficiency. The results suggest the acidophilic nature and thermostability property of Xyl-IIb from *Penicillium citrinum* isolate HZN13 which can be exploited for industrial applications. Xylanase from *Penicillium occitanis* has been reported to be active at pH 3.0 and 65 °C (Driss et al. 2012) and that from *Penicillium oxalicum* GZ-2 at pH 4.0 and 60 °C (Liao et al. 2014). Wakiyama et al. (Wakiyama et al. 2008) reported xylanase from *Penicillium citrinum* to be optimal at pH 6.0 and 50 °C and was stable at wide pH range and up to 40 °C.

#### Effect of metal ions, additives, surfactants, oxidizing agents and organic solvents

The effects of various metal ions on Xyl-IIb activity at 2, 5 and 10 mM concentrations are shown in Table 2. The metal ions such as Ca^2**+**^ and Ba^2+^ activated the enzyme Xyl-IIb, whereas Hg^2**+**^, Pb^2**+**^, Ni^2+^, Cu^2+^, and Cd^2+^ strongly inhibited Xyl-IIb at different concentrations. Our results are similar to those reported for xylanase from *Penicillium sclerotiorum* (Knob and Carmona 2010) and *Penicillium glabrum* (Knob et al. 2013). Sulfhydryl compounds, such as DTT, β-mercaptoethanol and cysteine, activated Xyl-IIb, whereas sulphydryl inhibitors, such as iodoacetamide and *p*-CMB, inhibited the enzyme activity (Table 3). These results suggest the involvement of thiol groups in the enzyme activity. 1,10-phenanthroline, a Fe^2+^-specific chelator, drastically inhibited enzyme activity indicating the presence of Fe^2+^ at its active site. Inhibition of enzyme by oxidizing agents like H_2_O_2_ may be due to oxidation of Fe^2+^ to Fe^3+^ in the enzyme. Similar findings have been reported from *Penicillium glabrum* (Knob and Carmona 2010).

**Table 2:** Effect of different metal ions on activity of purified Xyl-IIb

Metal ions	Relative activity (%)		
	2 mM	5 mM	10 mM
None	100	100	100
Co^2+^	112 ± 0.4	110 ± 0.5	98 ± 0.6
Zn^2+^	98 ± 0.3	90 ± 0.6	68 ± 0.5
Ca^2+^	122 ± 0.3	118 ± 0.7	104 ± 0.4
Mg^2+^	100 ± 0.6	97 ± 0.4	90 ± 0.6
Na^+^	110 ± 0.6	107 ± 0.4	100 ± 0.5
Cu^2+^	65 ± 0.4	50 ± 0.3	37 ± 0.4
Hg^2+^	30 ± 0.5	18 ± 0.2	–
Fe^3+^	90 ± 0.4	85 ± 0.5	70 ± 0.5
Pb^2+^	56 ± 0.3	45 ± 0.7	20 ± 0.6
Ni^2+^	50 ± 0.2	40 ± 0.6	18 ± 0.7
Mn^2+^	112 ± 0.1	106 ± 0.4	98 ± 0.8
Cd^2+^	35 ± 0.3	20 ± 0.5	–
Ba^2+^	128 ± 0.2	120 ± 0.4	110 ± 0.6

Enzyme assay carried out with 1 % (w/v) birchwood xylan in citrate buffer (pH 4.0) at 55 °C for 30 min in the absence of any metal ions was considered as 100 %. For the test samples, enzyme was pre-incubated with metal ions for 1 h prior to the assay

Experiments were carried out in triplicate and the results are presented as mean ± standard deviation (SD)

**Table 3:** Effect of various additives on activity of purified Xyl-IIb

Additives	Relative activity (%)		
	2 mM	5 mM	10 mM
None	100	100	100
DTT	122 ± 0.5	120 ± 0.2	106 ± 0.5
β-Mercaptoethanol	117 ± 0.6	111 ± 0.5	98 ± 0.6
l-Cysteine	166 ± 0.6	160 ± 0.4	150 ± 0.8
PMSF	65 ± 0.4	56 ± 0.5	30 ± 0.8
NBS	60 ± 0.5	50 ± 0.3	20 ± 0.9
Iodoacetamide	70 ± 0.2	68 ± 0.1	40 ± 0.6
*p*-CMB	68 ± 0.5	61 ± 0.6	45 ± 0.7
1,10-Phenanthroline	74 ± 0.7	68 ± 0.7	52 ± 0.4
H_2_O_2_	65 ± 0.6	47 ± 0.5	28 ± 0.7
SDS	125 ± 0.9	117 ± 1.1	98 ± 1.2
Tween-40	124 ± 0.7	122 ± 0.4	107 ± 0.7
Triton X-100	102 ± 0.4	93 ± 0.4	90 ± 0.4

Enzyme assay carried out with 1 % (w/v) birchwood xylan in citrate buffer (pH 4.0) at 55 °C for 30 min in the absence of any additives was considered as 100 %. For the test samples, enzyme was pre-incubated with additives for 1 h prior to the assay

Experiments were carried out in triplicate and the results are presented as mean ± SD

Xyl-IIb showed stability in the presence of detergents such as SDS. Xylanase from *Trichoderma inhamatum* (Silva et al. 2015) and *Penicillium glabrum* (Knob et al. 2013) have been reported to be inhibited by SDS. Surfactants like Tween-40 and Triton X-100 enhanced the enzyme activity at lower concentrations (Table 3). Surfactants have also been found to enhance the activity in *A. fumigates* Z5 (Miao et al. 2015).

Xyl-IIb showed a relative activity of greater than 85 % in most of the organic solvents like glycerol, ethanol, methanol, acetone, propanol, petroleum ether, isopropanol, benzene, cyclohexane, hexane, butanol and toluene at 30 % concentration except for formic acid which reduced the activity (Table 4). Solvent-tolerant property of enzymes is one of the important attributes of industrial enzymes. Solvent stability of xylanase from *A. tubingensis* FDHN1 has been reported (Adhyaru et al. 2015).

**Table 4:** Effect of different organic solvents on activity of purified Xyl-IIb

Organic solvent	Relative activity (%)		
	10 %	20 %	30 %
Glycerol	125 ± 1.2	114 ± 1.3	110 ± 0.9
Ethanol	98 ± 1.1	94 ± 1.2	90 ± 0.8
Methanol	110 ± 1.2	100 ± 1.4	96 ± 1.1
Acetone	96 ± 0.9	92 ± 0.6	88 ± 0.7
Formic acid	72 ± 0.9	64 ± 0.7	47 ± 0.8
Propanol	96 ± 0.8	94 ± 0.9	89 ± 0.9
Petroleum ether	94 ± 0.7	91 ± 1.1	90 ± 0.6
Isopropanol	100 ± 0.81	96 ± 0.7	92 ± 0.7
Benzene	96 ± 1.1	91 ± 0.8	87 ± 0.5
Cyclohexane	95 ± 1.2	90 ± 0.4	85 ± 0.4
Hexane	92 ± 0.9	85 ± 0.5	76 ± 0.7
Butanol	112 ± 0.7	97 ± 0.7	93 ± 0.8
Toluene	115 ± 0.5	98 ± 0.5	92 ± 0.4

Enzyme assay carried out with 1 % (w/v) birchwood xylan in citrate buffer (pH 4.0) at 55 °C for 30 min in the absence of any organic solvent was considered as 100 %. For the test samples, enzyme was pre-incubated with organic solvent for 1 h prior to the assay

Experiments were carried out in triplicate and the results are presented as mean ± SD

### Substrate specificity and kinetic studies

The purified GH-10 Xyl-IIb showed highest substrate specificity toward birchwood and oat spelts xylan with 6273 and 4818 U/mg of specific activities, respectively. GH-10 xylanases are commonly found to be active on a wide range of substrates unlike GH-11 family which shows specificity with few xylan substrates. No measurable activities were detected with other substrates indicating high substrate specificity and cellulase-free xylanase, which has potential application in pulp and paper industry. Kinetics of Xyl-IIb revealed a *K*_m_ of 10 and 16.7 mg/ml and *V*_max_ of 9523 and 15,873 U/mg with birchwood and oat spelts xylan, respectively. Comparatively, Xyl-IIb showed high affinity toward birchwood xylan. Differences in solubility and structure of substrates influence the activity and kinetic parameters. A high *K*_m_ and *V*_max_ is observed with oat spelts xylan. Enzyme interaction with oat spelts xylan might have varied the characteristic features of active site. Similarly, higher catalytic specificity for soluble birchwood xylan was also reported from *Trichoderma inhamatum* (Silva et al. 2015). GH-10 family XYN11A from *Penicillium oxalicum* GZ-2 showed highest specific activity with beechwood xylan (150.2 U/mg) in comparison to birchwood xylan (60.2 U/mg) and oat spelts xylan (72.6 U/mg) and also represented high affinity toward beechwood xylan (Liao et al. 2014).

### Cloning, sequence analysis, and expression of xynB gene (*Penicillium citrinum* isolate HZN13)

**Fig. 3:**
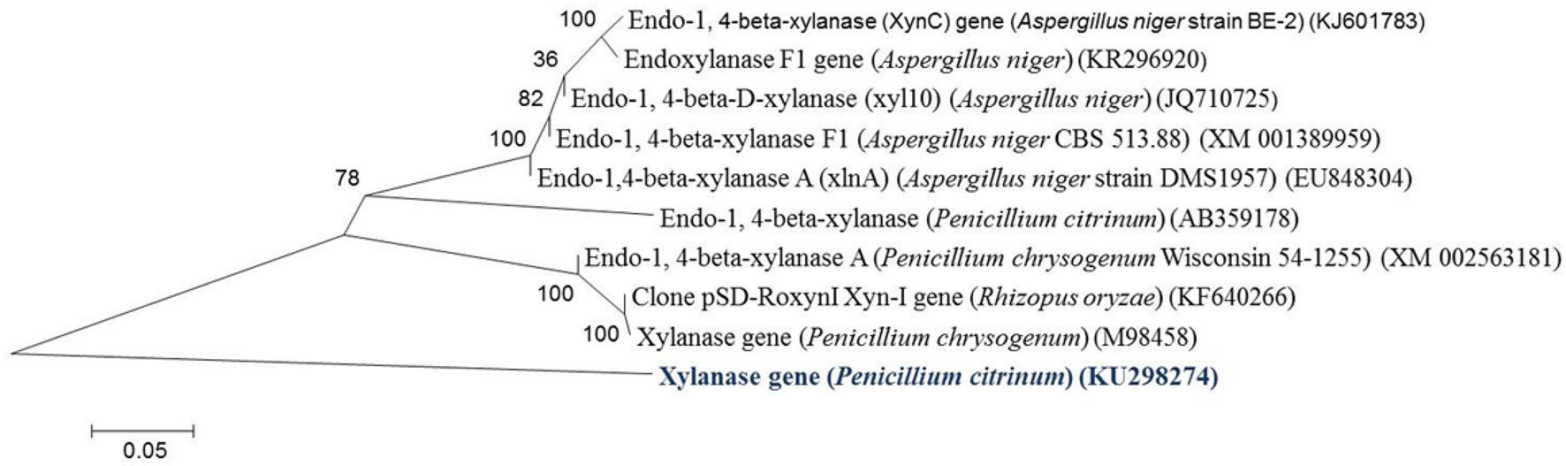
Phylogenetic relationships established by the neighbor-joining (NJ) method using xylanase gene (*xynB*) sequence of *Penicillium citrinum* isolate HZN13 with other related sequences. The *number* at *nodes* shows the bootstrap values obtained with 1000 resampling analyses

**Fig. 4:**
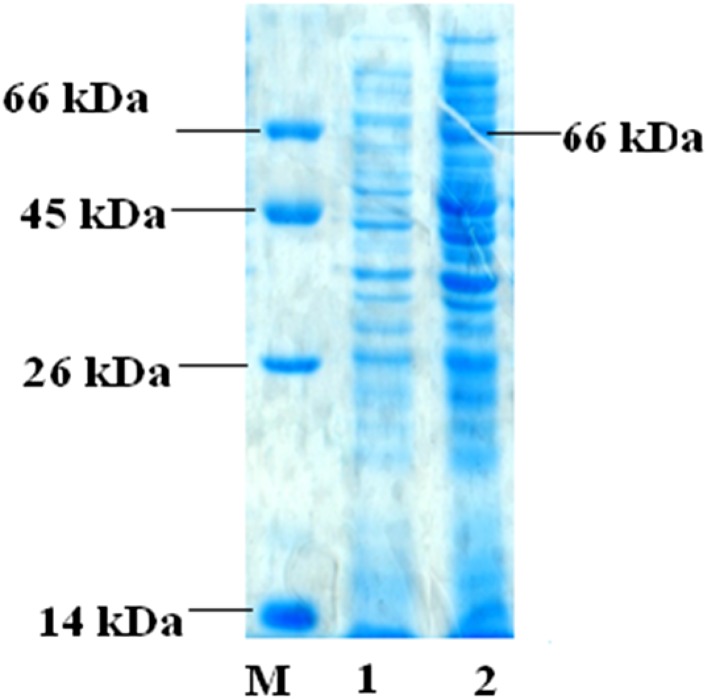
SDS-PAGE of proteins expressed in BL21 (DE3). *Lane M* four markers (66, 45, 26, 14 kDa). *Lane 1* uninduced clone in BL21 (DE3) and *Lane 2* induced clone in BL21 (DE3)

Using RT-PCR, a specific band of 1.5 kb was amplified from the total RNA of *Penicillium citrinum* isolate HZN13. The complete nucleotide sequence of *xynB* gene encoding any one of the xylanases (Xyl-I, Xyl-IIa, Xyl-IIb or Xyl-III) was determined. The nucleotide sequence was 1501 bp in length. Sequence analysis confirmed that the RT-PCR product had high homology to the members of the xylanase gene family. The phylogenetic tree was constructed using *Penicillium citrinum* isolate HZN13 *xynB* gene with other related xylanase genes (organisms) as shown in Fig. 3. Open reading frame (ORF) was located between nucleotides 1279 and 1542 with a 264-bp length of DNA sequence and is predicted to encode a protein of 87 amino acids. Fig. S2 shows a comparison of the putative amino acid sequence from *Penicillium citrinum* isolate HZN13 with those available on the databases and revealed close identity with endo-1,4-beta-xylanase from *Penicillium citrinum* (GenBank Accession Number BAG12101) with up to 37 (amino acid) base mutations. Although recombinant xylanase was highly similar to endo-1,4-beta-xylanase from *Penicillium citrinum*, it harbored different characteristic properties. The 37 base mutations are of great value for further study. The recombinant xylanase from *Penicillium citrinum* isolate HZN13 belonged to glycosyl hydrolase (GH) family 10 based on the highly conserved regions such as MVQIKAAAL, PRQASVSID, KFKAHGKKY, IKADF and NSMKWDATE. The deduced amino acid sequence of *xynB* gene was similar to the corresponding conserved region of fungal xylanases which have been classified into family GH-10. As previously reported, endo-1,4-beta-xylanase from *Penicillium citrinum* strain also belonged to GH-10 family with these conserved regions (Wakiyama et al. 2008). The cloned gene, the coding sequence of xylanase, was subcloned into the expression vector pGEX-4T-1 and expressed in *E. coli* BL21 (DE3) for verification. Extracellular xylanase activity of 80 U/mg was detected from the culture broth of the recombinant strain. A 66-kDa xylanase protein band from the cell-free extracts of *E. coli* BL21 (DE3) was shown on SDS-PAGE induced by IPTG, whereas in non-induced cell-free extracts of *E. coli* BL21 (DE3), no 66-kDa protein band was detected (Fig. 4). Xylanase was expressed as a fusion protein due to the presence of the glutathione S-transferase (GST)-tagged fusion peptide in expression vector pGEX-4T-1. Fusion protein with a molecular mass of 66 kDa corresponds to the sum of xylanase (40 kDa) and GST (26 kDa). From SDS-PAGE and zymogram analysis (Fig. 1), it can be concluded that *xynB* gene was successfully expressed as a Xyl-IIa protein in *E. coli* BL21 (DE3). These predictions are consistent with previously reported experimental data (Liao et al. 2015; Tanaka et al. 2005; Wakiyama et al. 2008).

## Conclusions

In the present study, the *Penicillium citrinum* isolate HZN13 produced a highly active cellulase-free GH-10 xylanase (Xyl-IIb). The purified Xyl-IIb was found to be solvent-thermostable–acidophilic in nature and *xynB* gene encoded xylanase (Xyl-IIa) which belonged to GH-10 family. Cloning and expression of *xynB* gene was successful in *E. coli* BL21 (DE3). The purified enzyme finds huge potential in biofuel and other industries.

## Electronic supplementary material

Below is the link to the electronic supplementary material.

Supplementary material 1 (DOCX 240 kb)
