# Supplementary material for: Purification, characterization, gene cloning and expression of GH-10 xylanase (Penicillium citrinum isolate HZN13)
Source: 3 Biotech. 2016 Aug 13;6(2):169. doi: 10.1007/s13205-016-0489-4 (PMC4987633; doi:10.1007/s13205-016-0489-4)
Supplement: Supplementary file 1 — Supplementary material 1 (DOCX 240 kb) [file 13205_2016_489_MOESM1_ESM.docx]

**Supplementary Information**

**Purification, characterization, gene cloning and expression of GH-10 xylanase (*Penicillium citrinum* isolate HZN13)**

Zabin K. Bagewadi, Sikandar I. Mulla and Harichandra Z. Ninnekar^*^

Department of Biochemistry, Karnatak University, Dharwad 580 003, Karnataka, India

Running title

**Purification of Xylanase from *Penicillium sp.***

^*^Corresponding author’s address: Dr. H Z. Ninnekar,

Professor,

Department of Biochemistry,

Karnatak University,

Dharwad 580 003, Karnataka, India.

Tel.: +91-0836-2215243; fax: +91-0836-2747884.

Email:[hzninnekar@yahoo.com](mailto:hzninnekar@yahoo.com)

**Supporting Information: 4 Pages, 2 Figures**

(A)

(B)

(C)

**Fig.S1.** Purification of xylanases from *Penicillium citrinum* isolate HZN13 by DEAE-Sepharose (A) Sephadex G-100 (B) and Biogel P-60 (C) chromatography. Arrow indicates pooled active fractions. Data values represent average of triplicates and error bars represent standard deviation.


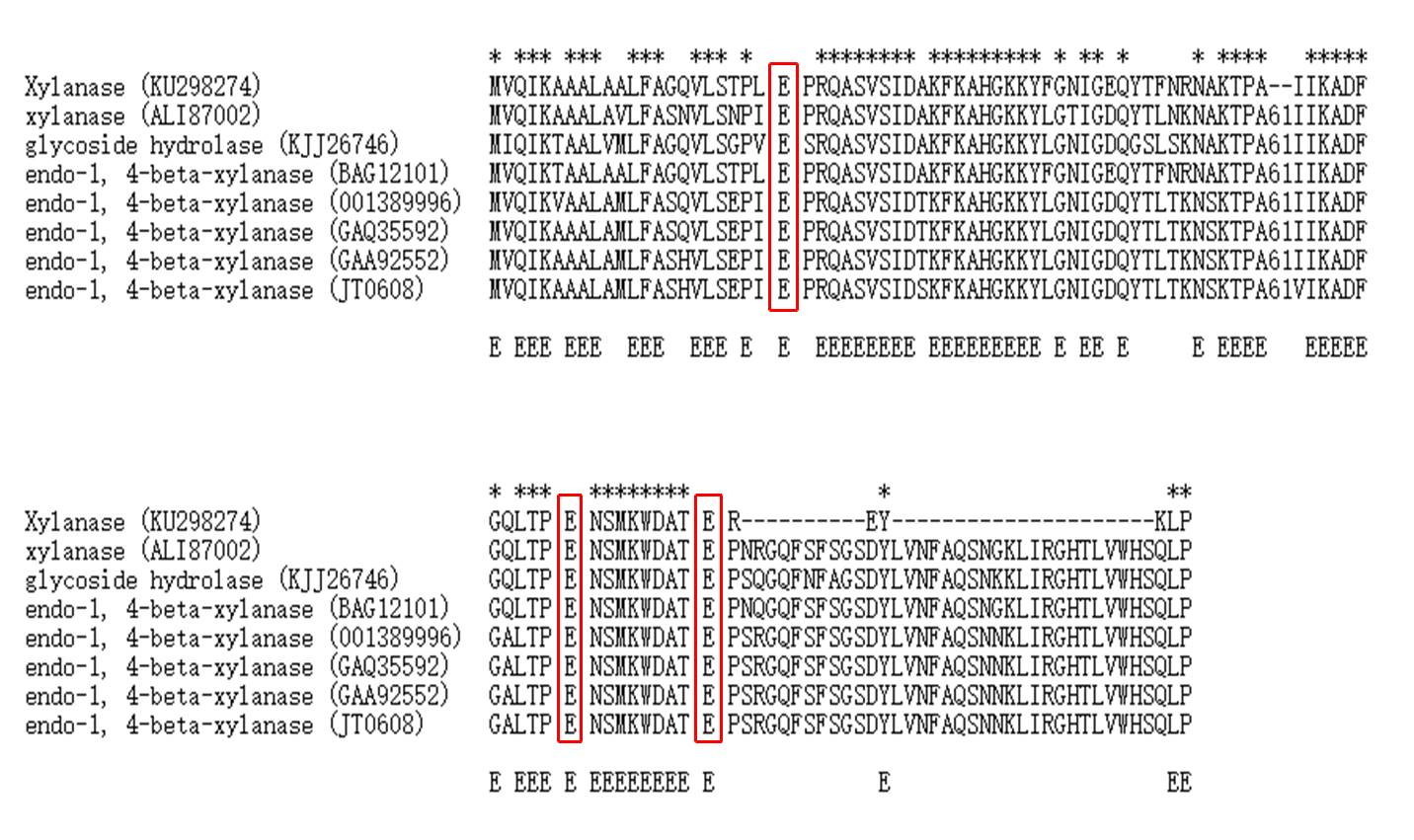


**Fig. S2.** Alignment of the predicted amino acid sequence of xylanase from *Penicillium citrinum* isolate HZN13 (GenBank accession Number KU298274) and its closest relatives from the GenBank databases. The closest sequences shown are xylanase from *Aspergillus aculeatus* (GenBank AN ALI87002); glycoside hydrolase from *Penicillium solitum* (GenBank AN KJJ26746); endo-1, 4-beta-xylanase from *Penicillium citrinum* (GenBank AN BAG12101); endo-1, 4-beta-xylanase from *Aspergillus niger* (GenBank AN 001389996); endo-1, 4-beta-xylanase from *Aspergillus niger* (GenBank AN GAQ35592); endo-1, 4-beta-xylanasefrom *Aspergillus kawachii* (GenBank AN GAA92552); and endo-1, 4-beta-xylanase from *Aspergillus niger* (GenBank AN JT0608). Gaps are showed by dashes. The catalytic residue is boxed, and residues marked E specify beta strands. Asterisks specify identical amino acids.
